# Supplementary material for: LTBP4, SPP1, and CD40 Variants: Genetic Modifiers of Duchenne Muscular Dystrophy Analyzed in Serbian Patients
Source: Genes (Basel). 2022 Aug 4;13(8):1385. doi: 10.3390/genes13081385 (PMC9407083; doi:10.3390/genes13081385)
Supplement: Supplementary file 1 [file genes-13-01385-s001.zip › genes-1815530-supplementary.pdf]

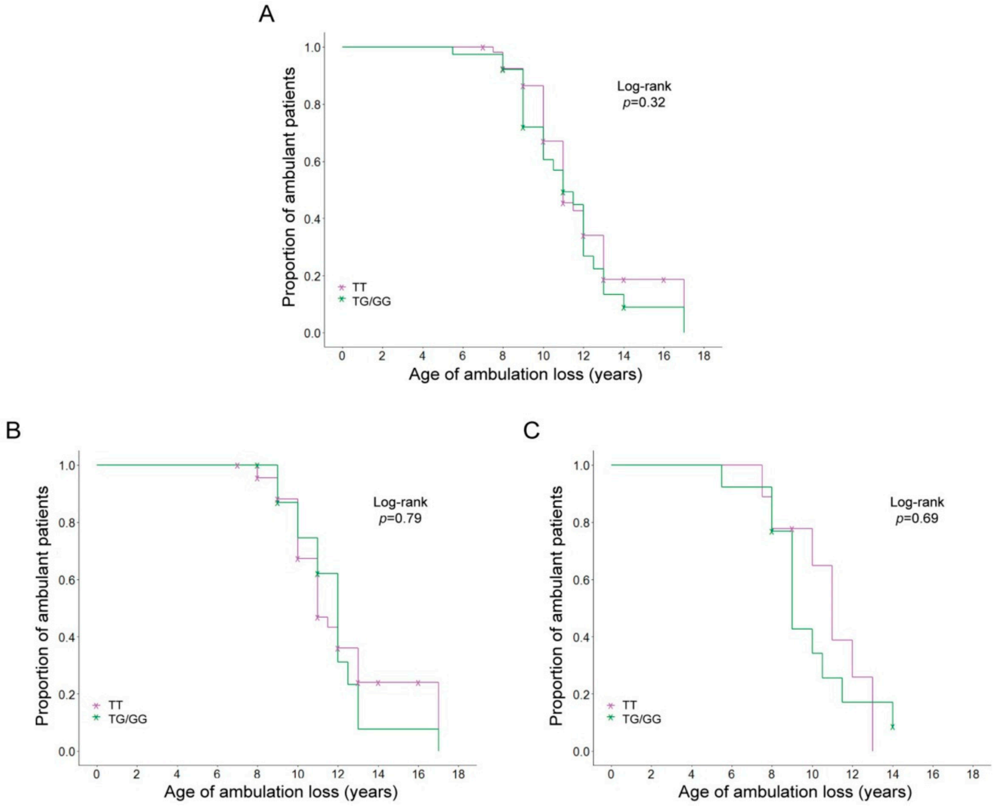

Figure S1. Kaplan–Meier plots showing the effect of rs28357094 in the *SPP1* gene on age at LoA for 93 patients with Duchenne muscular dystrophy. (**A**) The two survival lines represent patients stratified by the *SPP1* genotypes according to the dominant model (TT and TG/GG). Patients heterozygous or homozygous for a minor G allele showed no difference in age at LoA compared to patients homozygous for a T allele. The effect of the *SPP1* genotype was not significant neither in (**B**) GC treated, nor (**C**) in GC untreated patients. Log-rank test was used to compare different Kaplan-Meier curves and the corresponding p-values are shown on the top-right corner of all plots. Censored patients are indicated with an “x” on the survival lines.

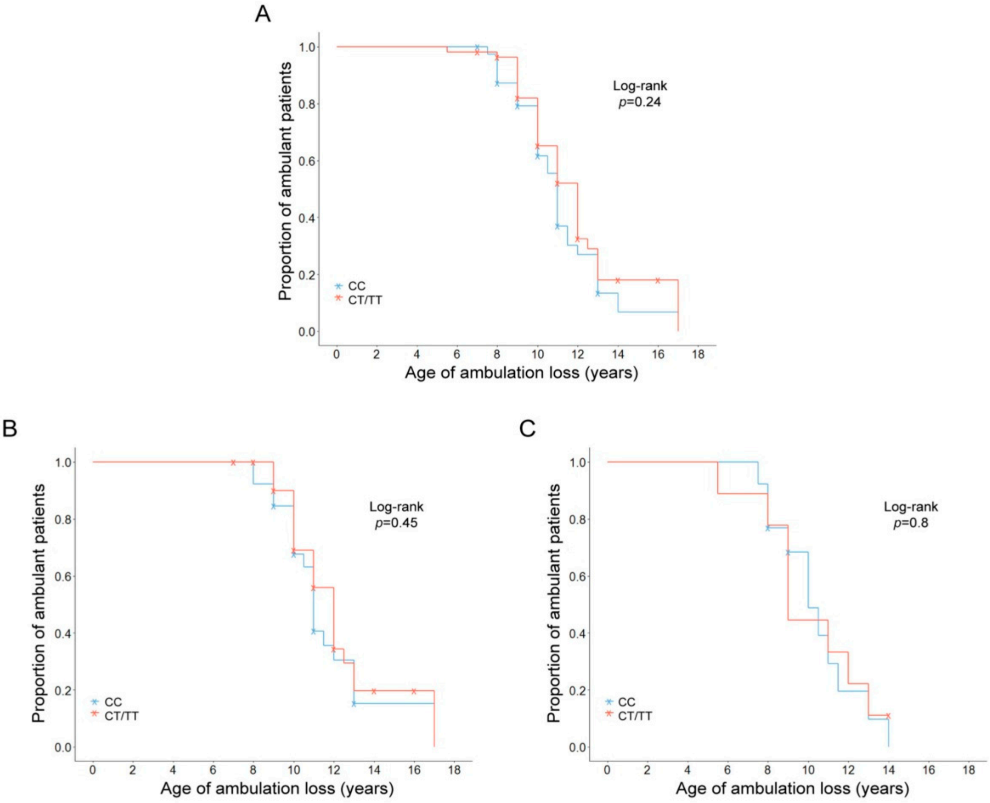

Figure S2. Kaplan–Meier plots showing the effect of rs1883832 in the *CD40* gene on age at LoA for 95 patients with Duchenne muscular dystrophy. (**A**) The two survival lines represent patients stratified by the *CD40* genotypes according to the dominant model (CC and CT/TT). Patients heterozygous or homozygous for a minor T allele showed no difference in age at LoA compared to patients homozygous for a C allele. The effect of the *CD40* genotype was not significant neither in (**B**) GC treated, nor (**C**) in GC untreated patients. Log-rank test was used to compare different Kaplan-Meier curves and the corresponding p-values are shown on the top-right corner of all plots. Censored patients are indicated with an “x” on the survival lines.

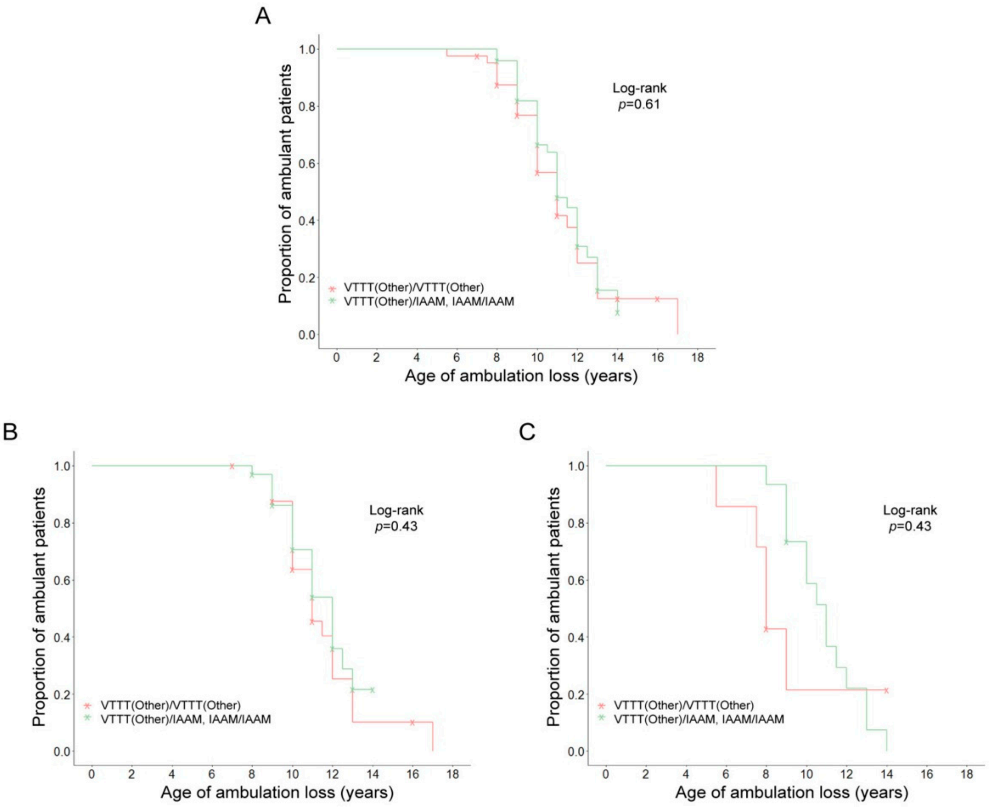

Figure S3. Kaplan–Meier plots showing the effect of the IAAM haplotype in the *LTBP4* gene (dominant model) on age at LoA for 89 patients with Duchenne muscular dystrophy. (**A**) Patients heterozygous or homozygous for the IAAM haplotype showed no difference in age at LoA compared to patients carrying other haplotypes. The effect of the *LTBP4* haplotype IAAM was not significant neither in (**B**) GC treated, nor (**C**) in GC untreated patients. Log-rank test was used to compare different Kaplan-Meier curves and the corresponding p-values are shown on the top-right corner of all plots. Censored patients are indicated with an “x” on the survival lines.

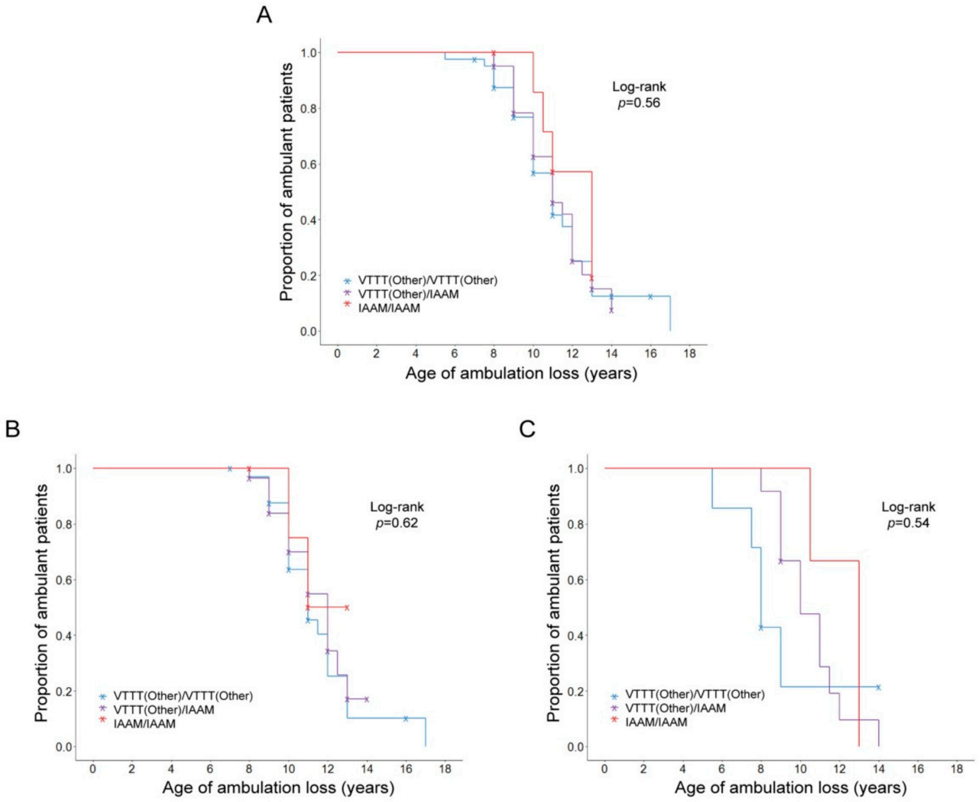

Figure S4. Kaplan–Meier plots showing the effect of the IAAM haplotype in the *LTBP4* gene (additive model) on age at LoA for 89 patients with Duchenne muscular dystrophy. (**A**) Patients heterozygous for the IAAM haplotype showed no difference in age at LoA compared to patients without the IAAM haplotype and no difference compared to patients homozygous for the IAAM haplotype. The effect of the *LTBP4* haplotype IAAM was not significant neither in (**B**) GC treated, nor (**C**) in GC untreated patients. Log-rank test was used to compare different Kaplan-Meier curves and the corresponding p-values are shown on the top-right corner of all plots. Censored patients are indicated with an “x” on the survival lines.

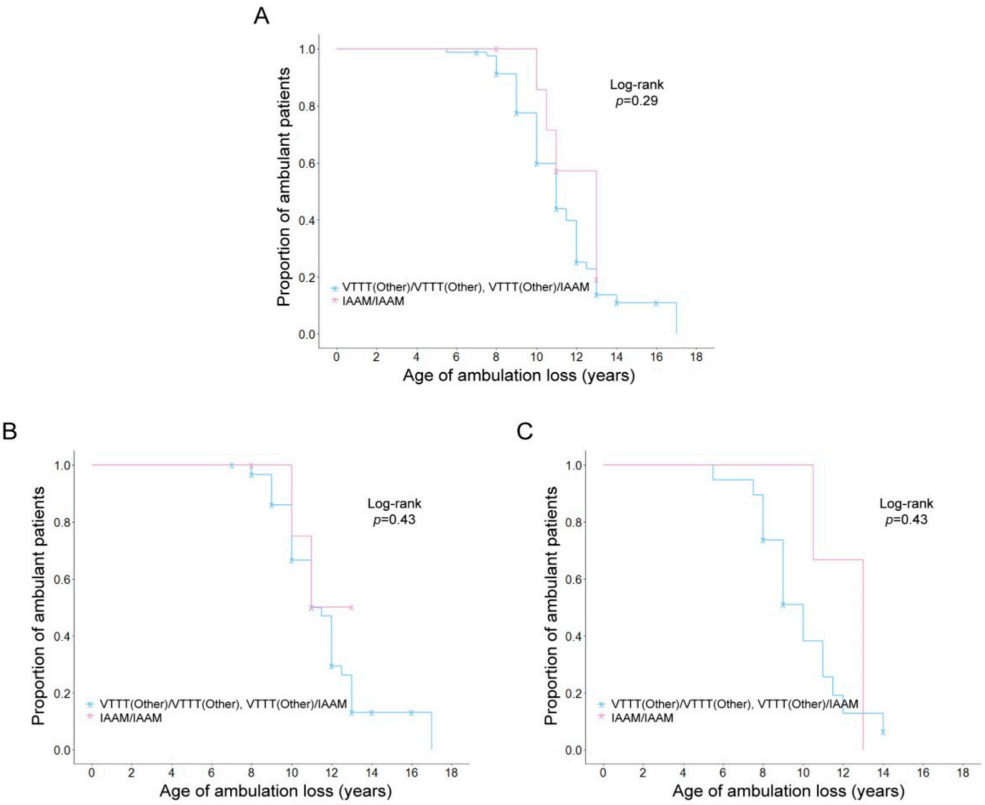

Figure S5. Kaplan–Meier plots showing the effect of the IAAM haplotype in the *LTBP4* gene (recessive model) on age at LoA for 89 patients with Duchenne muscular dystrophy. (**A**) Patients homozygous for the IAAM haplotype showed no difference in age at LoA compared to patients heterozygous for the IAAM or carrying any other haplotypes. The effect of the *LTBP4* haplotype IAAM was not significant neither in (**B**) GC treated, nor (**C**) in GC untreated patients. Log-rank test was used to compare different Kaplan-Meier curves and the corresponding p-values are shown on the top-right corner of all plots. Censored patients are indicated with an "x" on the survival lines.

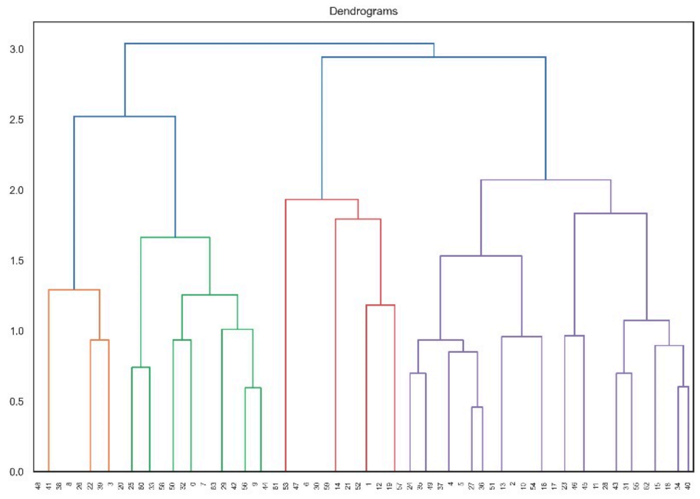

Figure S6. Dendrogram based on Wald's linkage hierarchical clustering method.
The vertical axis represents the measure of distance, indicated by the height of blue lines.
The 64 patients were clustered into four smallest defined clusters (shown in colors orange, green, red and purple) based on similarity. Additionally, two larger clusters can be observed that are comprised of smaller orange + green and red + purple clusters respectively, as indicated by the top dendrogram branch.
